# Therapeutic SERPINs: Improving on Nature

**DOI:** 10.3389/fcvm.2021.648349

**Published:** 2021-03-31

**Authors:** Coen Maas, Steven de Maat

**Affiliations:** CDL Research, University Medical Center Utrecht, Utrecht, Netherlands

**Keywords:** SERPIN, α1-antitrypsin, C1 esterase inhibitor, reactive center loop, therapy

## Abstract

Serine proteases drive important physiological processes such as coagulation, fibrinolysis, inflammation and angiogenesis. These proteases are controlled by serine protease inhibitors (SERPINs) that neutralize their activity. Currently, over 1,500 SERPINs are known in nature, but only 37 SERPINs are found in humans. Thirty of these are functional protease inhibitors. The inhibitory potential of SERPINs is in perfect balance with the proteolytic activities of its targets to enable physiological protease activity. Hence, SERPIN deficiency (either qualitative or quantitative) can lead to disease. Several SERPIN resupplementation strategies have been developed to treat SERPIN deficiencies, including concentrates derived from plasma and recombinant SERPINs. SERPINs usually inhibit multiple proteases, but only in their active state. Over the past decades, considerable insights have been acquired in the identification of SERPIN biological functions, their inhibitory mechanisms and specificity determinants. This paves the way for the development of therapeutic SERPINs. Through rational design, the inhibitory properties (selectivity and inhibitory potential) of SERPINs can be reformed and optimized. This review explores the current state of SERPIN engineering with a focus on reactive center loop modifications and backbone stabilization. We will discuss the lessons learned from these recombinant SERPINs and explore novel techniques and strategies that will be essential for the creation and application of the future generation of therapeutic SERPINs.

## Introduction

Approximately one third of all proteases belong to the superfamily of serine proteases, which can be found throughout all kingdoms of life. In humans, ~180 serine proteases govern essential physiological processes such as vascular hemostasis (1), inflammation (2), tissue remodeling (3) or angiogenesis (4). Many of these processes are regulated by chymotrypsin-like serine proteases, which are the most abundant class of serine proteases. These have a highly conserved proteolytic mechanism [reviewed in ((5))] and operate in “sequential activation” cascade mechanisms (e.g., coagulation or complement). The activity of serine proteases needs to be controlled, as excessive activity causes disease. This is where serine protease inhibitors (SERPINs) are of high importance.

### SERPINs in Human Physiology

The superfamily of SERPINs consists of ~1,500 identified members (6). There is evidence for the existence of 37 human SERPINs at protein level. Thirty of these have proven inhibitory function, where they act as suicide substrate inhibitors. Loss of SERPIN function can have severe pathological consequences. For example, patients with α1-antitrypsin (α1AT) deficiency develop pulmonary emphysema due to uncontrolled activity of neutrophil elastase (7). C1-esterase inhibitor (C1INH) deficiency leads to attacks of angioedema, due to excessive bradykinin formation by the plasma contact system (8), whereas patients with low levels of antithrombin (ATIII) have an increased risk of ischemic stroke, deep vein thrombosis or pulmonary embolism due to increased activity of the coagulation system (9). Currently, the majority of SERPIN therapeutics are meant as supplementation therapy to overcome these defects.

### SERPINs Mode of Action

**Figure 1:**
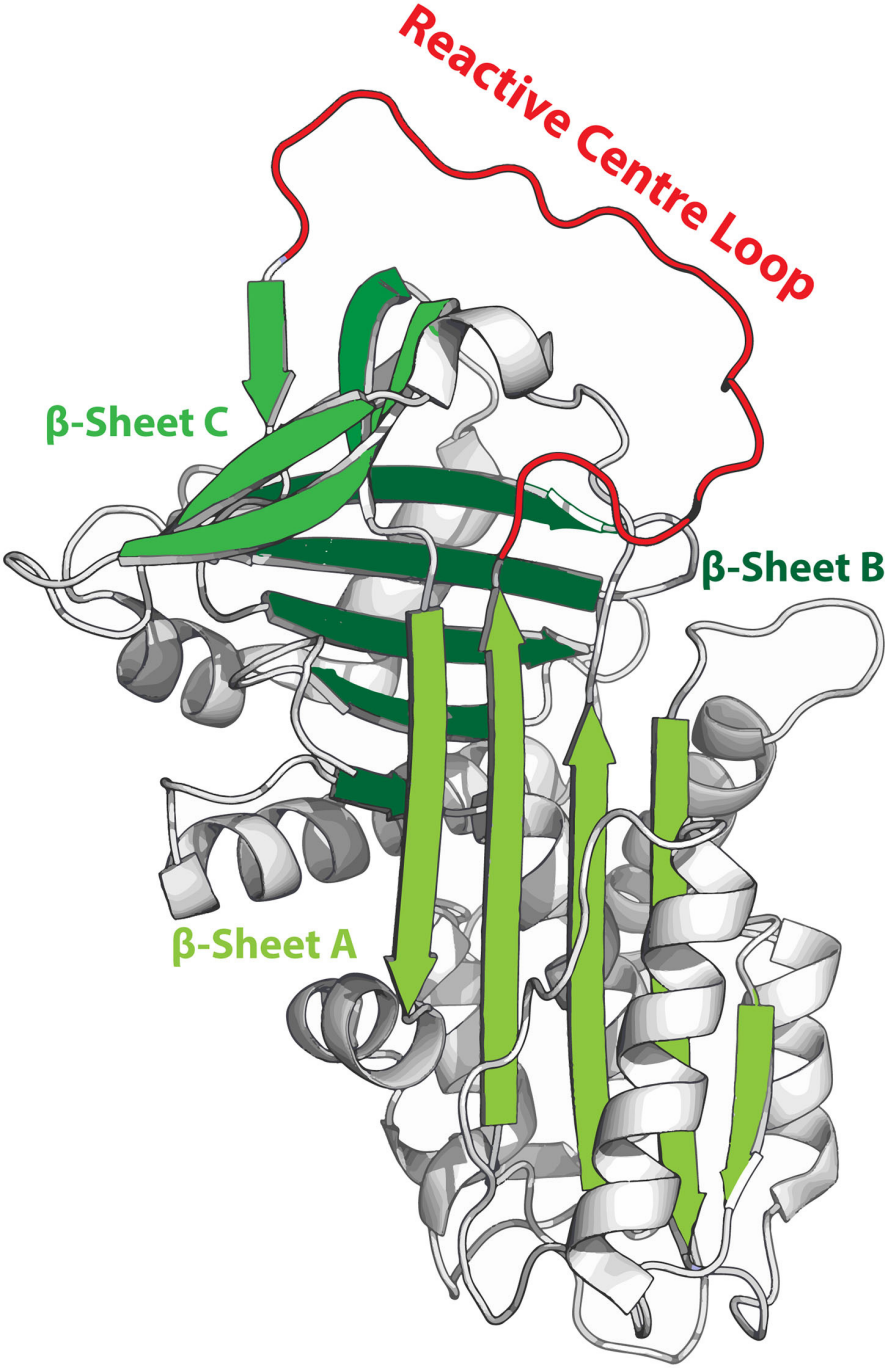
Metastable structure of α1-antitrypsin. β-sheets are highlighted in green and the reactive center loop (RCL) is highlighted in red. When the RCL becomes cleaved it integrates into β-sheet A, effectively becoming the 6th strand of this sheet.

SERPINS have a generally well-conserved secondary structure consisting of three β-sheets (A, B and C; highlighted in green in Figure 1) and nine α-helices (6, 10). Additionally, SERPINs contain an exposed reactive center loop (RCL; highlighted in red in Figure 1), which is a flexible loop structure on top of the SERPIN backbone. The RCL serves as a bait sequence for target proteases. The tertiary structure of native SERPINs is metastable, which can shift into a hyperstable conformation. This process is critical for the SERPIN function. While this shift can occur spontaneously, it becomes actively triggered when the RCL is cleaved by a protease (11). When the protease cleaves the RCL at the P1-P1'scissile bond, the serine (or a cysteine in case of cysteine proteases) of the catalytic triad of the protease attacks the carbonyl of the RCL, forming a tetrahedral intermediate (12). Hereafter, two situations may occur: (I) The SERPIN assumes its hyperstable state, pulling the protease to the opposite side of the SERPIN (13). Meanwhile, the N-terminal remainder of the RCL becomes inserted next to the five strands of β-sheet A, effectively making it the sixth strand (11, 14). During this process, the protease active site becomes distorted, and it can no longer hydrolyze the tetrahedral intermediate (15). When this occurs in the extracellular space, the SERPIN-protease complex will be cleared *via* scavenger receptors. II) The protease hydrolyzes the tetrahedral intermediate and releases it before active site disruption. The speed by which the C-terminal loop of the RCL is inserted into β-sheet A is critical and determines whether the SERPIN becomes a substrate or an inhibitor. Nonetheless, the SERPIN still folds into its hyperstable state (because its RCL has been successfully cleaved, which is irreversible) and will be rapidly cleared, while the regenerated protease remains active.

The ability of SERPINs to interact with multiple targets offers a unique opportunity for the therapeutic management of pathological enzyme systems. This review will explore the current state of SERPIN engineering, with a special focus on stabilizing SERPIN function and altering SERPIN specificity.

## Supercharging SERPINs

To supercharge SERPINs, co-factors can be administered to patients. Glycosaminoglycans can modulate the activity of several SERPINs by enhancing SERPIN functionality and therefore complex formation (16–21). For example, heparin (amongst others) enhances the efficiency of thrombin inhibition by endogenous antithrombin (from 7.2^*^10^3^ to 1.3^*^10^7^ M^−1^ · s^−1^ respectively) (22). More recently, polyphosphate was identified as a novel cofactor in the regulation of the complement system by enhancing the interaction between C1s and C1INH to an extent, similar to that of heparin (23). However, with the rise of recombinant protein technology, the doorway has been opened to change SERPINS for the treatment of disease states beyond SERPIN deficiency and can be used to control new therapeutic target proteases.

### Stability

Ideally, therapeutics are stable and in the case of life-long diseases should have a long half-life time in the human circulation. SERPINs are dependent upon their ability to shift from a metastable to a hyperstable conformation for function. This shift becomes problematic if it occurs spontaneously (i.e., without cleavage of the RCL by a target protease). This results in an inert SERPIN with the propensity to polymerize into pathogenic Z and S variants. Such behavior results in intracellular SERPIN accumulation and leads to tissue damage in the form of liver cirrhosis in the case of α1AT deficiency (24). Also for C1INH, intracellular accumulation has been reported for some mutations (25). However, there is little evidence to suggest that this is accompanied by liver cirrhosis. The differences in expression levels between both SERPINs are a logical explanation for this unwishful clinical phenotype. In order to avoid SERPIN polymerization during drug development, efforts are ongoing to achieve stabilization of the SERPIN backbone without it losing its inhibitory potential.

#### Lessons From Antitrypsin

Kwon et al. increased the thermostability of α1AT 13-fold by using a single mutation (F51C) without harming its inhibitory activity (26). Similarly, mutation F51L increased thermostability, but reduced the misfolding and polymerization of the pathogenic α1AT Z variant (27). Interestingly, the naturally-occurring (non-pathogenic) mutation F51S leads to α1AT retention in CHO-cells and reduces its stability (28). All the above mutations are at the same position: residue 51, demonstrating that subtle differences have a large impact on backbone stabilization (28). Further mutagenesis of the α1AT-F51L backbone identified six additional mutations (T59A, T68A. A70G, M374I, S381A) that improve α1AT stability without influencing inhibitory activity (29, 30). Other stabilizing mutations influenced the inhibitory capacity and are therefore less interesting for the development of therapeutic SERPINs (31).

#### Lessons From PAI-1

Not all SERPINs have a similar half-life and stability. Compared to other SERPINs, plasminogen activator I (PAI-1) has a short stability half-life of 1–2 h at 37°C and is considered relatively unstable (32). The binding of vitronectin to PAI-1 increases its stability 2–3 fold, but this complex would be considered unfavorable for therapeutic purposes. A single mutation (I91L) increases PAI-1 stability by nine-fold (32), whereas the combination of four mutations (N150H, K154T, Q319L and M354I) was able to increases the half-life 72-fold to 145 h (33). A set of 10 mutations (T50A, Q56R, A61V, G70D, T94A, N150D, D222G, I223V, G264D, and S331G) increases the stability even further to 540 h (34). Remarkably, the introduction of additional disulfide bridge (Cys 197–Cys 355) in the original PAI-1 backbone increases the stability to 700 h (35).

#### A Uniform Serpin Backbone

These results from work on α1AT and PAI-1 show the potential of engineering SERPIN stability, however these results cannot be directly extrapolated to other SERPIN molecules. To expedite the development of therapeutic SERPINs, attempts have been undertaken to create uniform SERPIN backbones that have been optimized for stability. Hereto, the group of Porebski et al. aligned the sequence of various SERPINs to identify their consensus sequence (36). The resulting molecule “Conserpin,” is stable up to temperatures of 110°C and shows resistance against polymerization. Furthermore, Conserpin is able to reversibly fold in response to chemical denaturation. Unfortunately, the inhibitory activity of Conserpin was found to be poor as it is unable to form stable covalent serpin-protease complexes (36, 37). While this behavior is improved by replacing nine amino acids of the Conserpin RCL (P7-P2') by that of α1AT, it still underperforms in comparison to wild type α1AT. As such, further insight in to SERPIN-protease interaction is required to allow therapeutic SERPINs to be optimized for stability without it affecting their efficacy. Although the RCL is very important, there are other motifs present in SERPINs that are important for target engagement such as exosites.

### Circulatory Half-Life

In humans, the circulatory half-life time between extracellular SERPINs differs quite significantly. For example, plasma-derived α1AT has a circulatory half-life of 4.5–8.7 days (38, 39). By comparison, its recombinant counterpart has a six-fold decrease in circulatory half-life (39, 40), which is thought to be the result of lacking, wrong or incomplete glycosylation. Although α1AT has been expressed in almost every host, the lack of proper glycosylation and circulatory half-life has been a major hurdle for any recombinant form of α1AT from reaching the market. Similar to α1AT, plasma-derived C1INH has a circulatory half-life of 22–56 h, but its recombinant variant (isolated from rabbit milk) only has a half-life of 2.4–3 h (41, 42). It is remarkable that prophylactic treatment with this molecule has therapeutic value (43), suggesting that it has biological properties unlike its natural counterpart which may facilitate alternative bio-distribution or cellular uptake which are beneficial to its therapeutic properties.

#### Enhanced Glycosylation

These obstacles have motivated efforts to optimize recombinant SERPIN glycosylation. Introduction of an additional N-glycosylation site (introduced *via* the Q9N mutation) was able to further increase the circulatory half-life of α1AT in rats (44). Recently, a modified CHO cell line was presented that delivered full humanized N-glycosylation profiles for both α1AT and the C1INH (45). Here, ten genes were knocked out to prevent glycosylation errors by the CHO cell line. Furthermore, the α-2,6-sialyltransferase enzyme (ST6GAL1) was overexpressed to improve capping of the N-glycans with alpha-2,6-linked sialic acid. While these recombinant variants of α1AT and C1INH exactly match their plasma derived counterparts when it comes to N-glycosylation profiles, their circulatory half-life times remain to be investigated.

#### Pegylation

To overcome the short circulation half-life of recombinant SERPINs, strategies have focused shielding the SERPIN *via* PEGylation (46–48). PEGylation of therapeutic proteins generally increases their biological stability and decreases their immunogenicity. Furthermore, PEGylation *via* a cysteine residue with an exposed thiol group (naturally present on certain SERPINs, including α1AT) is relatively straightforward and inexpensive. For recombinant α1AT, PEGylation did not influence its inhibitory potential *in vitro*, while pegylated α1AT variants (with 20 or 40 kDa PEG chains) showed increased circulation half-life, matching plasma-derived α1AT (48). Finally, in an *in vivo* elastase-mediated lung damage model, the PEGylated recombinant α1AT variants even outperformed its plasma derived counterpart.

#### Fusion Proteins

As an alternative approach to increase the circulation half-life, SERPINs have been fused to the Fc domains from IgG. This results in a homo-dimeric protein that should increase both efficacy and extend circulatory half-life (49). Currently, a phase I trial with α1AT-FC fusion protein (INBRX-101) is ongoing (https://clinicaltrials.gov/ct2/show/NCT03815396).

#### *In vivo* Expression

Trials with recombinant SERPINS are proven to be successful, patients would require weekly life-long therapy with injectables. As an attractive alternative, *in vivo* expression of SERPINs *via* gene therapy has been considered. For α1AT, both a phase I and II trial have been undertaken using a recombinant adeno-associated virus (AAV) vector which was administered to α1AT-deficient patients *via* intramuscular injection (50, 51). Patients tolerated the treatment and showed long term expression of α1AT. All subjects developed anti-AAV antibodies, but none developed antibodies against α1AT. While these studies confirmed the feasibility, patients only produced 20 μg/ml (0.38 μM) of α1AT in plasma serum, where therapeutic levels have to be at least 600 μg/ml (11.54 μM), where as normal levels are ~1.5 mg/ml (28.85 μM). While improved delivery of the gene therapy and improved SERPIN expression might help to overcome this problem, increasing the inhibitory activity of α1AT through mutagenesis might help to lower the levels that are required for therapy.

## Tailoring SERPIN Efficacy and Specificity

The RCL together with exosites are the major regulators of SERPIN activity. Exosites can directly improve the SERPIN-protease interaction, whereas the RCL sequence determines the SERPIN specificity by controlling which proteases active sites can cleave it. Even a single amino acid mutation in the RCL can have functional consequences. For example, wild-type α1AT is a potent inhibitor of neutrophil elastase, trypsin, chymotrypsin, tissue kallikrein 7 & 14, cathepsin G, neutrophil proteinase 3 and pancreatic elastase (Table 1), but not of coagulation proteases. Contrastingly, the RCL P1 mutation M358R (α1AT-Pittsburgh) converts it into a potent inhibitor of thrombin, activated protein C (APC), plasmin, factor XIa, factor Xa, plasma kallikrein and factor XIIa (Table 1). As a net result, patients with α1AT-Pittsburgh suffer from a life-long bleeding disorder (66, 67). This experiment of nature shows the impact of small RCL modifications.

**Table 1:** The Pittsburgh (M358R) mutation dramatically alters α1-antitrypsin specificity.

	**α1-Antitrypsin inhibition kinetics (*k*_2_: M^−1^·s^−1^)**			
	**Wild Type**		**Pittsburgh**	
Neutrophil Elastase	1.2-7*10^7^	(52, 53)		
Trypsin	2.8*10^5^	(53)		
Chymotrypsin	5.9*10^6^	(54)		
Tissue kallikrein 7	3.9*10^6^	(55)		
Tissue Kallikrein 14	2.6*10^5^	(56)		
Cathepsin G	4.1*10^5^	(57)		
Neutrophil proteinase 3	9.24*10^5^	(58)		
Pancreatic elastase	1.0*10^5^	(57)		
Thrombin	4.8*10^1^	(57)	2.9-3.6*10^5^	(59, 60)
Activated protein C	1.1*10^1^	(61)	0.49-1.1*10^5^	(59, 61)
Factor Xa	2.26*10^2^	(62)	4.13*10^4^	(59)
Factor XIa	6.6*10^1^	(63)	4-5.1*10^5^	(59, 63)
Plasmin	1.9*10^2^	(57)	2.5*10^6^	(64)
Plasma kallikrein	4.2	(63)	6.9-8.9*10^4^	(63, 65)
Factor XIIa	Not Detected	(63)	2.5-3.5*10^4^	(63, 65)

*K2: second-order rate inhibition constant*.

Despite its pathological nature, α1AT-Pittsburgh has been investigated as treatment for coagulopathy and mortality in sepsis. While α1AT-Pittsburgh treatment decreased mortality and coagulopathy was reported in a piglet sepsis model (68), a baboon model was unable to confirm these results and even showed signs of increased coagulopathy (69). The overall consensus was that the inhibition of APC and plasmin in this setting were unfavorable.

### Redesigning RCL Specificity

Various groups have attempted to refine the specificity of α1AT-Pittsburgh. Initial redesign of SERPIN specificity started as an “exchange program” by grafting RCL sequences onto different SERPINs backbones. This led to some success (70, 71); but was limited by the inhibitory behavior of the initial donor sequences. Although APC inhibition is considered unfavorable in the treatment of sepsis-related coagulopathy, Polderdijk et al. recently demonstrated that a refined α1AT variant (^357^KRK^359^), which selectively inhibits APC, has therapeutic value for the treatment of hemophilia A- and B (59). This molecule is currently in clinical development (https://www.clinicaltrials.gov/ct2/show/NCT04073498).

To unlock the true potential of SERPIN engineering for diverse diseases, further mutagenesis of the RCL is warranted. Yet, with each position that is mutagenized, the amount of total possibilities rises exponentially. Indeed, to fully mutagenize a sequence of eight amino acids (stretching the P4-P4' region) and use all 20 naturally occurring amino acids for, leads to a total of 2.56^*^10^10^ RCL sequences variants. Specific RCL positions have been thoroughly researched, which provides valuable information. For example, Schapira et al. showed that a single mutation helps to refine the inhibitory potential of α1AT-Pittsburgh (60). By altering the P2 position from a proline to an alanine (P357A) in α1AT-Pittsburgh, the inhibition of thrombin was diminished to the extent that it had no effects on the *ex vivo* thrombin time in plasma of Wistar rats. Interestingly, this ^357^AR^358^ mutation left the inhibition of FXIIa and PKa intact, protecting the rats in a model of bradykinin induced hypotensia. In 2002, Sulikowski et al. changed the RCL of α1AT-Pittsburgh into ^356^LGR^358^ or ^356^PFR^358^ to create a SERPIN to inhibit FXIIa, PKa and C1s (72). Where the ^356^LGR^358^mutant inhibited its designated targets, it also potently inhibited APC. By comparison, ^356^PFR^358^ showed an increase in specificity toward PKa. More recently, our group attempted to further improve the inhibition of the bradykinin producing proteases FXIIa and PKa. Based upon naturally occurring sequences and data from substrate peptide libraries, we created 18 α1AT variants. We found that only two new variants SMRT/V and SLLR/V (/ indicates RCL cleavage site) with a potent ability to inhibit FXIIa, PKa and FXIa, while showing negligible inhibition of thrombin, FXa and APC. These variants were effective in inflammatory models of carrageenan induced-paw swelling (driven by bradykinin) and dextran sulfate sodium-induced colitis as well as an injury-driven model of arterial thrombosis (73).

#### Peptide Libraries

While data from synthetic substrate peptide libraries (74) can be used to guide selection of lead RCL sequences, we experienced that data from these libraries unfortunately poorly translates into the wanted inhibitory behavior of full-length SERPINS (73). This probably relates to the non-linear structure of the RCL loop. To overcome this obstacle, others have performed high throughput SERPIN screening studies with the T7 phage display system (75, 76). While this method allowed to find thrombin inhibitors that are twice more potent that a1AT-Pittsburgh, their specificity toward other proteases remains to be investigated.

### Viral SERPINs

Like humans, viruses also express SERPINs to inhibit targets in their respective hosts (77). Examples of viruses that express SERPINs are the Orthopoxviruses, Myxoma viruses, Cowpox virus, Baculovirus and the Swinepox virus. These “cross-class” SERPINs enhance infection and suppress host inflammatory responses. Deletion of these SERPINs dramatically reduces the lethality rates, showing that these SERPINs act as virulence factors (78, 79). The Myxovirus expresses the SERPIN Serp-1, which inhibits urokinase plasminogen activator (uPA), tissue plasminogen activator, factor Xa, plasmin, and thrombin (in the presence of heparin). Serp-1 requires the uPA receptor to function *in vivo* (80–82). Interestingly, Serp-1 effectively suppresses arterial inflammation and plaque growth (83–85). In addition, a peptide mimicking the Serp-1 RCL showed therapeutic benefits in a MHV68 virus-induced vasculitis mouse model (86). However, the activity and stability of this peptide was different from full-length Serp-1. Protein modeling studies were performed to improve the inhibitory (and antiplaque) activity of Serp-1-based peptides. The resulting peptides indeed displayed increased inhibitory activity and were able to increase the survival rate of the mice in a MHV68 infection model of IFNγR KO mice (87). This work demonstrates the power of protein-modeling and shows its value for SERPIN design.

## Discussion

Over the past years, new molecular insights have been rapidly acquired that help recombinant SERPINs to fulfill their therapeutic promise. While the first recombinant SERPIN variants (the α1AT-FC fusion protein and the APC inhibiting α1AT variant) are moving into clinical development, the design of new SERPIN variants is still a very specialized and labor-intensive exercise. Improvements in molecular cloning strategies combined with protein modeling approaches will be of great importance to efficiently unlock the potential of SERPINs as therapeutic agents.

## Author Contributions

CM and SM wrote the manuscript. All authors contributed to the article and approved the submitted version.

## Conflict of Interest

The authors declare that the research was conducted in the absence of any commercial or financial relationships that could be construed as a potential conflict of interest.
